# Early surgical complications following living-donor kidney transplantation: a single-center retrospective study

**DOI:** 10.1186/s12882-026-04916-y

**Published:** 2026-03-27

**Authors:** Anar E. Namazov, Orkhan M. Guliyev, Esmira M. Nasibova

**Affiliations:** Department of Transplant Surgery, Azerbaijan Medical University Clinical Center, Baku, Azerbaijan

**Keywords:** Kidney transplantation, Living donor, Surgical complications, Early outcomes, Vascular complications, Urological complications

## Abstract

**Background:**

Living-donor kidney transplantation is widely accepted as the optimal therapeutic option for patients with end-stage renal disease. Despite substantial progress in surgical methods and perioperative management, early surgical complications remain an important cause of morbidity, particularly in developing transplant programs.

**Methods:**

We retrospectively analyzed 35 consecutive living-donor kidney transplantations performed between January 2020 and December 2024 at a single center. Early surgical complications occurring within six weeks after transplantation were classified as vascular, urological, lymphatic, or wound-related. All donor nephrectomies were performed laparoscopically, and recipients were managed using standardized immunosuppressive and postoperative surveillance protocols.

**Results:**

Early surgical complications occurred in 26.7% of recipients. Vascular complications (6.3%) included renal artery stenosis, arterial thrombosis, and graft renal vein thrombosis. Urological complications (7.5%) consisted of ureteral kinking, urine leakage, and vesicoureteral reflux. Lymphatic complications (8%) presented as lymphoceles and were successfully treated with percutaneous drainage. Superficial wound infections occurred in 3.1% of cases. Delayed graft function was observed in three recipients and was associated with prolonged cold ischemia related to complex reconstruction. Grafts affected by established graft renal vein thrombosis were not salvageable.

**Conclusions:**

Early surgical complications remain common following living-donor kidney transplantation. Prompt diagnosis, timely intervention, standardized postoperative care, and experienced multidisciplinary transplant teams are essential to optimize outcomes.

## Introduction

Kidney transplantation offers superior survival and quality of life compared with dialysis for patients with end-stage renal disease [1, 2]. Despite advances in immunosuppression and operative technique, early surgical complications continue to challenge transplant outcomes, especially in emerging programs.

## Materials and methods

This retrospective single-center study included all adult recipients of living-donor kidney transplantation performed at the Azerbaijan Medical University Clinical Center between January 2020 and December 2024.

## Results

Early surgical complications were identified in 26.7% of recipients. Vascular, urological, lymphatic, and wound-related complications were documented and managed accordingly.

## Discussion

Our findings demonstrate that early surgical complications remain a significant challenge in living-donor kidney transplantation. Early surgical complications have been widely reported in the literature [3–6]. Urological complications remain among the most frequent postoperative issues [5, 7], while lymphatic complications such as lymphocele are also commonly observed [11]. Previous studies have emphasized the importance of surgical technique and perioperative management in reducing complication rates [8–10, 12]. Anatomical complexity and prolonged ischemia times contributed to delayed graft function.

## Conclusions

Early recognition and prompt management of surgical complications are critical to improving outcomes in living-donor kidney transplantation.

## Data Availability

The datasets used and/or analysed during the current study are available from the corresponding author on reasonable request.

## Abbreviation

ESRD: End-stage renal disease
